# Prise en charge des instabilités patellaires objectives au Centre Hospitalier Universitaire Sylvanus Olympio de Lomé, Togo

**DOI:** 10.11604/pamj.2021.38.371.22956

**Published:** 2021-04-15

**Authors:** Tchaa Hodabalo Towoezim, Kolima Ehlissou Komlavi Akloa, Noufanague Kanfitine Kombate, Batarabadja Bakriga, Yaovi Yanick Dellanh, Grégoire Anani Abalo

**Affiliations:** 1 Service de Traumatologie-Orthopédie du Centre Hospitalier Universitaire (CHU) de Kara, Faculté des Sciences de la Santé de l´Université de Kara, Kara, Togo,; 2 Service de Traumatologie-Orthopédie du Centre Hospitalier Universitaire (CHU) Sylvanus Olympio de Lomé, Faculté des Sciences de la Santé de l´Université de Lomé, Lomé, Togo,; 3 Service de Traumatologie-Orthopédie de l´Hôpital Saint Jean de Dieu d´Afagnan, Faculté des Sciences de la Santé de l´Université de Lomé, Lomé, Togo

**Keywords:** Patella, instabilité, dysplasie de la trochlée, tubérosité tibiale antérieure, plastie d’Insall, ligament fémoro-patellaire médial, Togo, Patella, instability, trochlear dysplasia, anterior tibial tuberosity, Insall plasty, medial patellofemoral ligament, Togo

## Abstract

**Introduction:**

l´instabilité patellaire est une affection rare, multifactorielle et dont la prise en charge est complexe. Le but de notre étude était de décrire les cas d´instabilités patellaires, puis d´évaluer les résultats du traitement dans un pays à faible revenu.

**Méthodes:**

l´étude s´est déroulée de mars 2013 à février 2018. Elle a porté sur huit patients qui avaient un âge supérieur à 15 ans et qui avaient été traités chirurgicalement pour instabilité patellaire objective. La fonction du genou avant et après le traitement chirurgical a été appréciée par le score IKDC.

**Résultats:**

l´âge moyen lors de la chirurgie était de 28,5 ans et le sexe féminin prédominait avec six cas. La dysplasie de la trochlée a été le principal facteur étiologique retrouvé chez sept patients. Dans quatre cas cette dysplasie était associée à la hauteur de la patella avec l´index de Caton moyen de 1,4. Dans cinq cas, il a été réalisé la section du retinaculum patellaire latéral associée à la transposition de la TTA et à la plastie d´Insall. L´évaluation fonctionnelle du traitement a été satisfaisante, avec un score IKDC moyen de 91,3%.

**Conclusion:**

les instabilités patellaires sont rares. Leur prise en charge est retardée dans nos pays en voie de développement, mais les résultats sont bons si les protocoles sont adaptés.

## Abstract (en)

**Introduction:**

patellar instability is a rare multifactorial condition whose treatment is complex. The purpose of this study was to describe cases of patellar instabilities and then to assess treatment outcomes in low-income countries.

**Methods:**

the study was conducted from March 2013 to February 2018. Eight patients aged >15 years and who had undergone surgery for objective patellar instability were enrolled. Before and after surgery knee function was assessed using IKDC score.

**Results:**

the average age at surgery was 28.5 years and female sex was predominant (6 cases). Trochlear dysplasia was the main etiological factor (7 patients). In four cases dysplasia was related to patellar height (mean Caton index: 1.4). In five cases, section of lateral patellar retinaculum associated with TTA transposition and Insall’s plasty was performed. Functional outcome was satisfactory, with a mean IKDC score of 91.3%.

**Conclusion:**

patellar instabilities are rare. Management is delayed in our developing countries, but outcomes are good when protocols are adapted.

## Introduction

L´instabilité patellaire concerne souvent des patients jeunes et actifs [1]. Elle est rare, avec une incidence estimée à 1 pour 1000 personnes [2]. A cause de son étiologie qui est multifactorielle, son diagnostic et son traitement restent un défi pour le chirurgien. Pour une première luxation de la rotule, le traitement est conservateur, sauf en présence de lésions intra articulaires nécessitant un geste chirurgical. En cas d´instabilité rotulienne récidivante, le traitement chirurgical se fait à la carte, s´adressant aux facteurs d´instabilités identifiés [3]. En Afrique subsaharienne, l´instabilité patellaire fait rarement l´objet de consultation. Les rares patients qui consultent sont vus souvent à un âge adulte, lorsque le genou devient invalidant. Notre étude avait pour objectif d´exposer les résultats de la prise en charge de cette affection grave dans un pays subsaharien.

## Méthodes

**Type et cadre d´étude**: il s´est agi d´une étude prospective réalisée au centre hospitalier universitaire Sylvanus Olympio de Lomé au Togo, sur une période de cinq ans, de mars 2013 à février 2018.

**Population étudiée**: l´étude a porté sur les patients qui avaient été traités pour instabilité patellaire. Nous avons retenu huit cas pour lesquels les critères d´inclusion étaient: l´âge supérieur à 15 ans, avec une luxation latérale de la patella survenue plus d´une fois, et qui ont été opérés après un bilan radiologique qui a confirmé le diagnostic d´instabilité patellaire objective. Nous avons exclu de l´étude les patients perdus de vue.

**Collecte des données**: les patients étaient sélectionnés lors des consultations et les données ont été recueillies sur une fiche d´enquête pré établie, pendant les différentes phases de la prise en charge. Les paramètres étudiés étaient l´âge, le sexe, la fonction du genou avant la chirurgie, les résultats du bilan paraclinique, les différents protocoles opératoires et les résultats fonctionnels du genou après la chirurgie.

**Bilan radiologique et techniques chirurgicales**: le bilan morphologique a permis d´étudier la dysplasie de la trochlée selon la classification de H. Dejour [4], la hauteur de la patella évaluée selon l´index de Caton-Deschamps [5], la bascule latérale de la patella, la distance entre la tubérosité antérieure et la gorge de la trochlée (TA-GT) et la nature du ligament fémoro-patellaire médial (LFPM). Sur le plan chirurgical, plusieurs gestes ont été réalisés: la transposition de la tubérosité tibiale antérieure (TTA) a été le seul geste osseux. Elle a consisté en une ostéotomie de la TTA, en sa médialisation et son abaissement selon les valeurs de la distance TA-GT et la hauteur de la patella. Les gestes sur les parties molles ont regroupé la section extra articulaire du retinaculum patellaire latéral, la plastie d´Insall qui est l´abaissement et la latéralisation du muscle vaste médial, et la plastie du ligament fémoro-patellaire médial (LFPM) par les ischio-jambiers. Pour les cas où le cartilage de croissance était présent, la médialisation du tendon patellaire a été faite selon la technique de la baguette molle.

**Evaluation fonctionnelle du traitement chirurgical**: elle a été faite après un recul moyen de 48 mois grâce au score IKDC (International Knee Documentation Committee) [6].

**Considération éthique**: tous les patients adultes et les parents des mineurs avaient donné leur consentement éclairé avant leur intégration à l´étude.

## Résultats

**Aspects démographiques**: en cinq ans, nous avons traité 8 patients. L´âge moyen à la consultation était de 28,5 ans avec des extrêmes de 16 et de 48. L´âge moyen du début des symptômes était de 19 ans avec des extrêmes qui étaient de 8 et de 46. Le sexe féminin était prédominant avec six cas et la sex ration F/H= 3 (Tableau 1).

**Tableau 1:** aspects sociodémographiques et mode d´apparition de l´instabilité patellaire

N°	Age	Sexe	Durée de la symptomatologie avant la consultation	Côté	Gêne fonctionnel	1^er^ épisode
1	20	F	4 ans	G	Chutes	Traumatisme mineur
2	48	M	3 ans	D	Blocage	Choc violent
3	21	F	6 ans	D	Douleurs, chutes	Spontané, lors de la marche
4	28	F	2 ans	D	Douleur	Traumatisme mineur
5	16	F	4 ans	D	Blocage	Traumatisme mineur
6	33	F	22 ans	D	Blocage	Spontané au réveil
7	46	M	27 ans	G	Douleur	Traumatisme mineur
8	16	F	5 ans	G	Douleur, blocage	Traumatisme mineur

**Aspects cliniques et radiologiques**: le motif de consultation pour tous les patients a été le déplacement latéral et récidivant de la patella. La douleur était la gêne la plus représentée (6 cas). Dans 5 cas, le 1^er^ épisode de luxation était dû à un traumatisme mineur (Tableau 1). Les signes physiques ont été dominés par la mobilité transversale anormale de la patella qui était positive dans tous les cas. Sur le plan fonctionnel, le score IKDC préthérapeutique moyen était de 45,11% avec des extrêmes qui étaient de 28,73% et de 57,47%. Il y avait sept cas de dysplasie de la trochlée. La patella était haute dans quatre cas, avec l´index de Caton-Deschamps moyen qui était de 1,4. Il y avait une gonarthrose fémoro patellaire chez trois patients. La Tomodensitométrie avait révélé une distance TA-GT supérieure à 20 mm dans 5 cas (Tableau 2).

**Tableau 2:** bilan radiologique et diagnostique

	Stade de la dysplasie	Hauteur de la patella Icd	Distance TA-GT	MPFL	Type de luxation
1	D	1,6	22	Rupture du LFPM et rétinaculum	Episodique
2	B	0,9	20	Rupture du LFPM et rétinaculum	Episodique
3	Dysplasie de la patella	1,1	21mm	Rupture du LFPM et rétinaculum	Habituelle
4	D	2,1	21mm	Rupture du LFPM et rétinaculum	Episodique
5	B	1,2	20,5	Rupture du LFPM et rétinaculum	Episodique
6	D	1,6	22mm	Rupture du LFPM et rétinaculum	Habituelle
7	D	1,8	19	Rupture du LFPM et rétinaculum	Episodique
8	B	1,1	19	Rupture du LFPM et rétinaculum	Episodique

**Figure 1:**
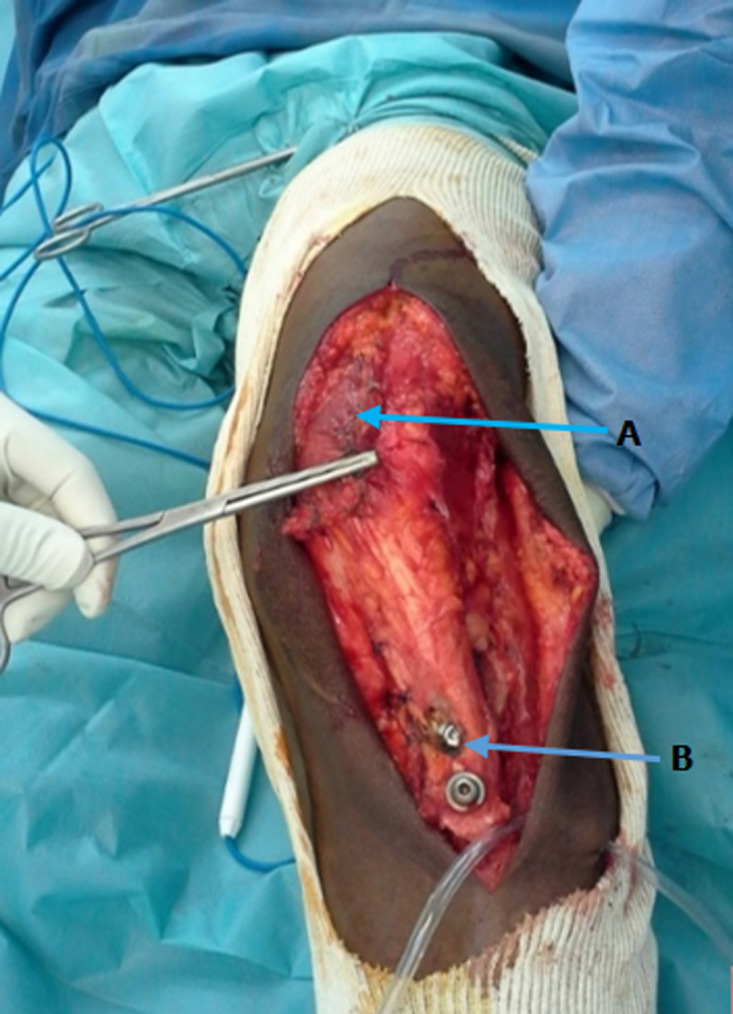
plastie d’Insall et transfert de la TTA, patient de 46 ans; A) abaissement et latéralisation du muscle vaste médial; B) abaissement et médialisation de la TTA maintenue par deux vis

**Traitement et évolution**: le protocole chirurgical associant trois gestes, notamment la section extra articulaire du retinaculum patellaire latérale, la transposition de la TTA, et la plastie d´Insall, a été le plus utilisé (Figure 1). La rééducation du genou a débuté après six semaines post opératoire dans tous les cas. Les résultats sur le plan anatomique ont montré que la TTA a consolidé en 3 mois en moyenne, l´index de Carton-Deschamps moyen était de 1,1 avec des extrêmes de 0,9 et de 1,3. Sur le plan fonctionnel, il a été noté un cas de récidive. Les résultats avant et après le traitement sont regroupés dans le Tableau 3.

**Tableau 3:** tableau récapitulatif de la technique chirurgicale et de la fonction du genou avant et après le traitement

N°	IKDC initial	Technique chirurgicale	IKDC Final	Récidive
1	57,47%	Section du rétinaculum patellaire latéral + transposition de la TTA + plastie d´insall	91,95%	Non
2	39,08%	Section du rétinaculum patellaire latéral + transposition de la TTA + plastie d´insall	89,65%	Non
3	28,73%	Section du rétinaculum patellaire latéral + transposition de la TTA + plastie d´insall	80,46%	Non
4	51,72%	Section du rétinaculum patellaire latéral + transposition de la TTA + plastie d´insall	98,85%	Non
5	54,02%	Section du rétinaculum latéral + médialisation du ligament patellaire + plastie du MPFL	95,40%	Non
6	35,63%	Section du rétinaculum latéral + rétente du rétinaculum médial	72%	Oui: engagement tardif de la patella
7	42,53%	Section du rétinaculum patellaire latéral + transposition de la TTA + plastie d´insall	80,5%	Non
8	51,72%	Section du rétinaculum latéral + médialisation du ligament patellaire + plastie du MPFL	98%	Non

## Discussion

Nous avons mené une étude prospective qui a porté sur une affection grave, mais peu fréquente, retrouvée en majorité dans la population jeune et féminine. Il s´agit d´adolescents actifs, et selon Tscholl *et al*. l´âge de prédilection se situerait entre 10 et 17 ans [1]. Dans notre étude, l´âge moyen d´apparition des premiers signes était de 19 ans mais la prise en charge a été faite à un âge moyen de 28,5 ans. Ceci s´explique par la tolérance de la société vis-à-vis de certaines maladies qui n´engagent pas le pronostic vital, mais aussi par le manque de moyens financiers, d´où une consultation tardive lorsque le genou devient invalidant avec parfois des complications telles que l´arthrose.

La dysplasie de la trochlée a été le facteur anatomique le plus retrouvé dans notre étude, suivie par la distance TA-GT excessive et la patella alta. Selon plusieurs auteurs, l´étiologie de l´instabilité patellaire est multifactorielle et la dysplasie trochléaire est un facteur de risque important à la luxation rotulienne récurrente. De plus avec la patella alta, l´engagement dans la trochlée ne se produit pas dans la phase précoce de la flexion du genou, potentialisant ainsi l´instabilité fémoropatellaire. Les mêmes auteurs évoquent également parmi les facteurs étiologiques le *genu valgum*, une antéversion fémorale accrue, une torsion externe du tibia et/ou une tubérosité tibiale latéralisée qui peuvent tous affecter la stabilité de la patella [7]. Le retard de prise en charge de cette affection a entrainé une gonarthrose précoce dans trois cas. Heikki *et al*. ont rapporté dans leur étude que 35% des patients ayant été opérés tardivement de l´instabilité patellaire ont développé une arthrose précoce fémoropatellaire [8].

La chirurgie à la carte a été faite, associant plusieurs gestes en trois protocoles. L´association de la section du retinaculum latéral et la retente du retinaculum médial s´est révélée insuffisante, à l´origine de la récidive que nous avons notée. En effet la remise en tension des éléments musculo-ligamentaires médiaux dans le traitement chirurgical de l´instabilité patellaire oppose, outre la section du retinaculum latéral, la plastie d´Insall associée à la retente du rétinaculum médial d´une part, et la plastie du LFPM d´autre part. Et d´après Kulkarni *et al*. la plastie du LFPM aurait de meilleurs résultats [9]. Mais il est recommandé que ces techniques sur les parties molles se fassent en association avec les gestes osseux que sont la transposition de la TTA selon la distance TA-GT et la hauteur de la patella, ou la trochléoplastie chez l´adulte. La trochléoplastie est contre indiquée en présence de cartilage de croissance ou d´arthrose fémoro-patellaire sévère. Si elle est considérée comme pourvoyeuse d´arthrose par plusieurs auteurs, Otsuki *et al*. rapportent de bons résultats pour des patients âgés de plus de 40 ans et présentant des dysplasies trochléennes de type B et D selon Dejour, associées à une patella alta [10]. Cette technique pourra donc être utilisée dans notre situation où les patients sont pris en charge à un âge avancé. Nos résultats au dernier recul ont été satisfaisants, le score IKCD moyen était favorable, à 91,3%. Otsuki *et al*. ont retrouvé au dernier récul 86,7% et 73,3% respectivement selon les scores Lysholm et kudjala, après un protocole associant la trochléoplastie [10].

## Conclusion

Les résultats issus du traitement chirurgical des instabilités patellaires objectives dépendent des protocoles qui doivent être adaptés à chaque patient selon les anomalies identifiées. La rigueur et la maitrise des différents gestes sont donc requises chez les opérateurs. Les efforts doivent être faits dans nos pays pour une prise en charge précoces avant l´apparition des complications.

### Etat des connaissances sur le sujet

- Pathologie multifactorielle, dominées par la dysplasie de la trochlée;
- La reconstruction du ligament fémoro-patellaire médiale est la technique la plus pratiquée actuellement, en association avec d´autres gestes.

### Contribution de notre étude à la connaissance

- Rapporter le retard de prise en charge chirurgicale des instabilités patellaires en Afrique, l´âge moyen de prise en charge étant de 28,5 ans;
- Tenir compte de la présence de complications telle que l´arthrose dans la planification chirurgicale.
